# Supplementary material for: Loss of Ena/VASP interferes with lamellipodium architecture, motility and integrin-dependent adhesion
Source: eLife. 2020 May 11;9:e55351. doi: 10.7554/eLife.55351 (PMC7239657; doi:10.7554/eLife.55351)
Supplement: Supplementary file 1. [file elife-55351-supp1.docx]

| **Key Resources Table** | | | | |
| --- | --- | --- | --- | --- |
| **Reagent type (species) or resource** | **Designation** | **Source or reference** | **Identifiers** | **Additional information** |
| gene (*Mus musculus*) | *Vasp* | NIH 3T3 cDNA | MGI:  109268 | (isoform 1) |
| gene (*Mus musculus*) | *Evl* | NIH 3T3 cDNA | MGI:  1194884 | (isoform 2) |
| gene (*Mus musculus*) | *Enah* | NIH 3T3 cDNA | MGI:  108360 | (isoform 2) |
| Cell line (*Mus musculus*) | B16-F1 | ATCC | CRL-6323 | Mouse melanoma cells (Wild type) |
| Cell line (*Mus musculus*) | E-KO #23 | This study |  | Mouse melanoma B16-F1 derived Evl-KO cells |
| Cell line (*Mus musculus*) | EV-KO #23.7 | This study |  | Mouse melanoma B16-F1 derived Evl/VASP-KO cells |
| Cell line (*Mus musculus*) | EVM-KO #23.7.66 | This study |  | Mouse melanoma B16-F1 derived Evl/VASP/Mena-KO cells |
| Cell line (*Mus musculus*) | E-KO #27 | This study |  | Mouse melanoma B16-F1 derived Evl-KO cells |
| Cell line (*Mus musculus*) | EV-KO #27.9 | This study |  | Mouse melanoma B16-F1 derived Evl/VASP-KO cells |
| Cell line (*Mus musculus*) | EVM-KO #27.9.12 | This study |  | Mouse melanoma B16-F1 derived Evl/VASP/Mena-KO cells |
| Cell line (*Mus musculus*) | MV^D7^ | Bear et al., 2000 |  | Mouse embryonal fibroblasts  Ena/VASP-deficient;  immortalized with SV40 wild-type large T antigen |
| Cell line (*Mus musculus*) | MVE-KO #18 | This study |  | Mouse embryonal fibroblasts  MV^D7^ derived  Evl-KO cells |
| Cell line (*Mus musculus*) | MVE-KO #31 | This study |  | Mouse embryonal fibroblasts  MV^D7^ derived  Evl-KO cells |
| Cell line (*Mus musculus*) | NIH 3T3 | ATCC | CRL-1658 | Mouse embryonal fibroblasts  /Wild type) |
| Cell line (*Mus musculus*) | NIH 3T3 | ATCC | CRL-1658 | Mouse embryonal fibroblasts |
| Cell line (*Mus musculus*) | M-KO #10 | This study |  | Mouse embryonal fibroblasts  NIH 3T3 derived Mena-KO cells |
| Cell line (*Mus musculus*) | M-KO #13 | This study |  | Mouse embryonal fibroblasts  NIH 3T3 derived Mena-KO cells |
| Cell line (*Mus musculus*) | MV-KO #10.6 | This study |  | Mouse embryonal fibroblasts  NIH 3T3 derived Mena/VASP-KO cells |
| Cell line (*Mus musculus*) | MV-KO #10.15 | This study |  | Mouse embryonal fibroblasts  NIH 3T3 derived Mena/VASP-KO cells |
| Transfected construct (*Mus musculus*) | pEGFP-C1-Evl | This Study |  | Evl rescue |
| Transfected construct (*Mus musculus*) | pEGFP-C1-VASP | This Study |  | VASP rescue |
| Transfected construct (*Mus musculus*) | pEGFP-C1-Mena | This Study |  | Mena rescue |
| Transfected construct (*Mus musculus*) | pmCherry-C1-VASP | This Study |  | VASP rescue  in fluorescence time-laps microscopy |
| Transfected construct (*Homo sapiens*) | pEGFP-C1-Fascin | Adams & Schwartz, 2000 |  |  |
| Transfected construct (*S. cerevisiae*) | pEGFP-C1-Lifeact | Riedl et al., 2008 |  |  |
| Transfected construct (*Homo sapiens*) | pEGFP-C1-β-actin | Clontech | #6116-1 |  |
| Transfected construct (*Bos taurus*) | pEGFP-C1-MyoX | Berg & Cheney, 2002 |  |  |
| Transfected construct (*Homo sapiens*) | pEGFP-C1-Rif-L77 | Aspenström et al., 2004 |  |  |
| Transfected construct (*Mus musculus*) | pEGFP-C1-FMNL2-E272 | Kage et al, 2017 |  |  |
| Transfected construct (*Mus musculus*) | pEGFP-C1-FMNL3-E275 | Kage et al, 2017 |  |  |
| Transfected construct (*Mus musculus*) | pEGFP-C1-mDia2ΔDAD | Block et al., 2008 |  |  |
| Antibody | Rabbit polyclonal  anti-Evl | This study |  | WB: (1:1000) |
| Antibody | Rabbit polyclonal  anti-VASP | This study |  | WB: (1:1000) |
| Antibody | Rabbit polyclonal  anti-Mena | This study |  | WB: (1:1000) |
| Antibody | Rabbit polyclonal  anti-WAVE2 | This study |  | WB: (1:1000)  IF: (1:1000) |
| Antibody | Rabbit polyclonal  anti-GFP | Faix et al., 2001 |  | WB: (1:1000) |
| Antibody | Mouse monoclonal  anti-GAPDH | Merck | #CB1001-500UG | WB: (1:1000) |
| Antibody | Polyclonal goat anti-Mouse IgG (H+L) Alc. Phosphatase | Dianova | #115-055-62 | WB: (1:1000) |
| Antibody | Polyclonal goat anti-Rabbit IgG (H+L) Alc. Phosphatase | Dianova | #115-055-144 | WB: (1:1000) |
| Antibody | Rabbit polyclonal  anti-Cortactin | This study |  | IF: (1:1000) |
| Antibody | Mouse monoclonal  anti-Fascin 5E2 | This study |  | WB & IF: Undiluted Hybridoma supernatant |
| Antibody | Mouse monoclonal  anti-p16-ARCA 323H3 | Obazabal et al., 2003 |  | IF: Undiluted Hybridoma supernatant |
| Antibody | Mouse monoclonal  anti-Vinculin | Sigma-Aldrich | #V9131 | IF: (1:1000)  hVIN-1 |
| Antibody | Mouse monoclonal  anti-capping protein α1/α2 subunits | Developmental Hybridoma Bank, University of Iowa, Iowa City, IA | #mAb 5B12.3 | IF: Undiluted Hybridoma supernatant |
| Antibody | Polyclonal goat anti-Mouse IgG (H+L) Alexa Fluor® 488 | Invitrogen | #A-11029 | IF: (1:1000) |
| Antibody | Polyclonal goat anti-Mouse IgG (H+L) Alexa Fluor® 555 | Invitrogen | #A-32727 | IF: (1:1000) |
| Antibody | Polyclonal goat anti-Rabbit IgG (H+L) Alexa Fluor® 488 | Invitrogen | #A-11034 | IF: (1:1000) |
| Antibody | Polyclonal goat anti-Rabbit IgG (H+L) Alexa Fluor® 555 | Invitrogen | #A-21429 | IF: (1:1000) |
| Recombinant DNA reagent | pEGFP-C1 | Clontech | #U55763.1 |  |
| Recombinant DNA reagent | pmCherry | Addgene | ID: 632524 |  |
| Recombinant DNA reagent | pPur | Clontech | #631601 |  |
| Recombinant DNA reagent | pSpCas9(BB)-2A-Puro | Addgene | #48139 | Used for generating CRISPR/Cas9-constructs |
| Recombinant DNA reagent | pGEX-6P1 | GE Healthcare | #28-9546-48 | Used for generating constructs for recombinant proteins |
| Recombinant DNA reagent | pGEX-6P1- murine Evl | This study |  | Used for generating recombinant murine Evl (1-414) |
| Recombinant DNA reagent | pGEX-6P1- VASP | This Study |  | Used for generating recombinant murine VASP (1-375) |
| Recombinant DNA reagent | pGEX-6P1- Mena | This Study |  | Used for generating recombinant murine Mena (1-241) |
| Recombinant DNA reagent | pGEX-6P1- WAVE2-VCA (419-497) | This Study |  | Used for generating recombinant murine WAVE2-VCA antigen (419-497) |
| Recombinant DNA reagent | pGEX-6P2-cortactin | Lai et al., 2009 |  | Used for generating constructs for recombinant cortactin antigen |
| Sequence-based reagent | Cas9-targeting sequence Evl | This study | Cas9-targeting sequence | GATCGGTACCCACTTCTTAC |
| Sequence-based reagent | Cas9-targeting sequence VASP | This study | Cas9-targeting sequence | GTAGATCTGGACGCGGCTGA |
| Sequence-based reagent | Cas9-targeting sequence Mena | This study | Cas9-targeting sequence | AAGGGAGCACGTGGAGCGGC |
| Sequence-based reagent | pSpCas9 seq | This study | Sequencing primer | GGACTATCATATGCTTACCG |
| Sequence-based reagent | Evl target seq for | This study | CRISPR target PCR primer | AAGCCATGAGTCTCCCAAGC |
| Sequence-based reagent | Evl target seq rev | This study | CRISPR target PCR primer | GTCTCACGCTTTGGCTCTCA |
| Sequence-based reagent | VASP target seq for | This study | CRISPR target PCR primer | GTGTGGCCTGCCTATCTGTT |
| Sequence-based reagent | VASP target seq rev | This study | CRISPR target PCR primer | CAGAGGGACAGAGGGACAGA |
| Sequence-based reagent | Mena target seq for | This study | CRISPR target PCR primer | TCAGGCAACTGCAAGAACAG |
| Sequence-based reagent | Mena target seq rev | This study | CRISPR target PCR primer | CATCTCGGCTGTAGGAGGTG |
| Peptide, recombinant protein | murine Evl (1-414) | This Study |  | Antigen for immunization |
| Peptide, recombinant protein | murine VASP (1-375) | This Study |  | Antigen for immunization |
| Peptide, recombinant protein | murine Mena (1-241) | This Study |  | Antigen for immunization |
| Peptide, recombinant protein | WAVE2-VCA (419-497) | This Study |  | Antigen for immunization |
| Commercial assay or kit | VenorGeM Mycoplasma Detection Kit | Sigma | # MP0025 | Mycoplasma detection |
| Commercial assay or kit | Pierce BCA Protein Assay Kit | Thermo Scientific | 23227 |  |
| Commercial assay or kit | JetPrime | Polyplus | #114-07 | Transfection |
| Chemical compound, drug | ATTO-555 phalloidin | ATTO-TEC | #AD 550-82 | Visualization of actin cytoskeleton |
| Chemical compound, drug | EGFP-nanobodies Alexa Fluor® 488 | Chromotek | #gba488 | Enhancing EGFP signal |
| Chemical compound, drug | CK666 | Sigma | #SML0006-5MG | Arp2/3 complex inhibitor |
| chemical compound, drug | BCIP | CarlRoth | #6368.2 |  |
| chemical compound, drug | Benzonase | Merck | #71205-3 |  |
| chemical compound, drug | isopropyl-ß-D-thiogalactoside (IPTG) | CarlRoth | #2316.5 |  |
| chemical compound, drug | Protino glutathione-conjugated agarose 4B | Macherey-Nagel | #11912302 |  |
| chemical compound, drug | PreScission Protease | GE Healthcare | #GE27-0843-01 |  |
| chemical compound, drug | Paraformaldehyde | AppliChem | #A3813,0250 |  |
| Chemical compound, drug | Laminin | Sigma-Aldrich | #L2020 | Surface coating |
| Chemical compound, drug | Fibronectin | Roche | #11051407001 | Surface coating |
| Chemical compound, drug | Matrigel | Corning | #356234 | 3D nvasion assay |
| Software, algorithm | MetaMorph | Molecular Devices Corp. |  |  |
| Software, algorithm | Origin 2018G | OriginLab |  |  |
| Software, algorithm | Sigma plot 12.0 | Systat Software GmbH |  |  |
| Software, algorithm | GraphPad Prism 5 | GraphPad |  |  |
| Software, algorithm | Excel 2010 | Microsoft |  |  |
| Software, algorithm | Photoshop CS6 | Adobe |  |  |
| Software, algorithm | CorelDraw Graphics Suite 6X | CorelDraw |  |  |
| Software, algorithm | FAAS | Berginski et al., 2013 |  |  |
| Software, algorithm | Cell migration analysis macro | Litschko et al., 2018 |  |  |
