# Supplementary material for: Loss of Ena/VASP interferes with lamellipodium architecture, motility and integrin-dependent adhesion
Source: eLife. 2020 May 11;9:e55351. doi: 10.7554/eLife.55351 (PMC7239657; doi:10.7554/eLife.55351)
Supplement: Supplementary file 2. [file elife-55351-supp2.docx]

**Sequencing Results and Tide analysis of Ena/VASP mutants**

guide target site

**B16-F1 mouse melanoma cells**

**B16-F1 WT (Evl target sequence)**

ATGGTCTACGATGACACCAGTAAGAAGTGGGTACCGATCAAGCCTGGCCAGCAGGGATTCAGCCGGATCAACATCTACCACAACACTGCCAGCAGCACCTTCAGAGTGGTCGGGGTCAAGCTACAGGACCAGCAG

E-KO #23 allele 1 🡪 98 bp deletion 🡪 frame shift

ATGGTCTACGATGACACCAGTAA---------------------------------------------------------------------------------------------------TACAGGAC

CAGCA

E-KO #23 allele 1 🡪 1 insertion 79 bp deletion 🡪 frame shift

ATGGTCTACGATGACACCAGTAA**A**GAAGTGGGTACCGATCAA--------------------------------------------------------------------------------TACAGGAC

CAGCAG

E-KO #27 alleles 1 and 2🡪 7 bp deletion 🡪 frame shift

ATGGTCTACGATGACACCA-------GTGGGTACCGATCAAGCCTGGCCAGCAGGGATTCAGCCG

GATCAACATCTACCACAACACTGCCAGCAGCACCTTCAGAGTGGTCGGGGTCAAGCTACAGGACCAGCAG

**B16-F1 WT (VASP target sequence)**

CGAGACGGTCATCTGTTCCAGCCGGGCTACTGTGATGCTTTATGATGACAGCAACAAGCGATGGCTCCCTGCTGGCACTGGTCCCCAGGCCTTCAGCCGCGTCCAGATCTACCACAACCCCACTGCTAACTCCTTCCGAGTTGTTGGCCGCAAGATGCAGCCGGATCAGCAG

EV-KO #23.7 allele 1 🡪 1 bp insertion, 7 bp deletion 🡪 frame shift

CGAGACGGTCATCTGTTCCAGCCGGGCTACTGTGATGCTTTATGATGACAGCAACAAGCGATGGCTCCCTGCTGGCACTGGTCCCCAGGCCTTCAG**G**CCGCGTCCAGATCTACCACAACCCCACTGCTAACTCC-------TTGTTGGCCGCAAGATGCAGCCGGATCAGCAG

EV-KO #23.7 allele 2 🡪 1 bp insertion, 7 bp deletion 🡪 frame shift

CGAGACGGTCATCTGTTCCAGCCGGGCTACTGTGATGCTTTATGATGACAGCAACAAGCGATGGCTCCCTGCTGGCACTGGTCCCCAGGCCTTCAG**G**CCGCGTCCAGATCTACCACAACCCCACTGCTAACTCC-------TTGTTGGCCGCAAGATGCAGCCGGATCAGCAG

EV-KO #27.9 allele 1🡪 13 bp deletion 🡪 frame shift

CGAGACGGTCATCTGTTCCAGCCGGGCTACTGTGATGCTTTATGATGACAGCAACAAGCGATGGCTCCCTGCTGGCACTGGTCCCCAGGCCT------------GATCTACCACAACCCCACTGCTAACT

CCTTCCGAGTTGTTGGCCGCAAGATGCAGCCGGATCAGCAG

EV-KO #27.9 allele 2 🡪 7 bp deletion 🡪 frame shift

CGAGACGGTCATCTGTTCCAGCCGGGCTACTGTGATGCTTTATGATGACAGCAACAAGCGATGGCTCCCTGCTGGCACTGGTCCCCAGGCC-------GCGTCCAGATCTACCACAACCCCACTGCTAAC

TCCTTCCGAGTTGTTGGCCGCAAGATGCAGCCGGATCAGCAG

**B16-F1 (Mena WT target sequence)**

GCAACTGCAAGAACAGCAGCGACAGAAGGAACTGGAGAGGGAAAGAATGGAGAGGGAAAGGTTGGAGAGAGAACGACTAGAACGAGAGAGGCTAGAGAGGGAGCGCCTGGAACAAGAGCAGCTGGAGCGGCAGCGGCAGGAAAGGGAGCACGTGGAGCGGCTGGAGAGGGAGAGGCTGGAGCGCCTGGAGCGAGAGAGGCAGGAGCGGGAGCGAGAGCGCCTGGAGCAGCTGGAGCGGGAGCAAGTGGAGTGGGAGCGAGAGCGCAGAATGTCCAATGCTG

EVM-KO #23.7.66 allele 1🡪 1 bp insertion 🡪 frame shift

GCAACTGCAAGAACAGCAGCGACAGAAGGAACTGGAGAGGGAAAGAATGGAGAGGGAAAGGTTGGAGAGAGAACGACTAGAACGAGAGAGGCTAGAGAGGGAGCGCCTGGAACAAGAGCAGCTGGAGCGGCAGCGGCAGGAAAGGGAGCACGTGGAGC**A**GGCTGGAGAGGGAGAGGCTGGAGCGCCTGAGCGAGG

AGAGGCAGGAGCGGGAGCGAGAGCGCCTGGAGCAGCTGGAGCGGGAGCAAGTGGAGTGGGAGCGAGAGCGCAGAATGTCCAATGCTG

EVM-KO #23.7.66 allele 2🡪 1 bp insertion 🡪 frame shift

GCAACTGCAAGAACAGCAGCGACAGAAGGAACTGGAGAGGGAAAGAATGGAGAGGGAAAGGTTGGAGAGAGAACGACTAGAACGAGAGAGGCTAGAGAGGGAGCGCCTGGAACAAGAGCAGCTGGAGCGGCAGCGGCAGGAAAGGGAGCACGTGGAGC**C**GGCTGGAGAGGGAGAGGCTGGAGCGCCTGGAGCGAGAGAGGCAGGAGCGGGAGCGAGAGCGCCTGGAGCAGCTGGAGCGGGAGCAAGTGGAGTGGGAGCGAGAGCGCAGAATGTCCAATGCTG

EVM-KO #27.9.12 allele 1🡪5 bp deletion 🡪 frame shift

GTTGTGATAAACTGTGCCATTCCTAAAGGGCTGAAGTACAATCAAGCTACACAGACTTTCCACCAATGGAGGGATGCTAGACAGGTGTATGGTCTCAACTTTGGCAGCAAAGAGGATGC-----TCTTCG

CAAGTGCCATGATGCATGCCTTAGAAGTGTTAAATTCACAGGAAGCA

EVM-KO #27.9.12 allele 2🡪 19 bp deletion 🡪 frame shift

GTTGTGATAAACTGTGCCATTCCTAAAGGGCTGAAGTACAATCAAGCTACACAGACTTTCCACCAATGGAGGGATGCTAGACAGGTGTATGGTCTCAACTTTGGC-------------------TCTTCG

CAAGTGCCATGATGCATGCCTTAGAAGTGTTAAATTCACAGGAAGCA

**********************************

**MV^D7^ fibroblasts**

**MV^D7^ (Evl target sequence)**

ATGGTCTACGATGACACCAGTAAGAAGTGGGTACCGATCAAGCCTGGCCAGCAGGGATTCAGCCGGATCAACATCTACCACAACACTGCCAGCAGCACCTTCAGAGTGGTCGGGGTCAAGCTACAGGACCAGCAG

MVE-KO #18 allele 1 🡪 1bp insertion 5 bp deletion 🡪 frame shift

ATGGTCTACGATGACACCAGTAA**A**GAAGTGGGTACCGATCAAGCCTGGCCAGCAGGGATTCAGCCGGATCAACATCTACCACAACACTGCCAGCAGCACCTTCAGAGTGGTCGGGGTCA-----CAGGAC

CAGCAG

MVE-KO #18 allele 2🡪2 bp deletion, 1 bp insertion 5 bp deletion 🡪 frame shift

ATGGTCTACGATGACACCA--**A**AAGAAGTGGGTACCGATCAAGCCTGGCCAGCAGGGATTCAGCC

GGATCAACATCTACCACAACACTGCCAGCAGCACCTTCAGAGTGGTCGGGGTT-----TACAGGA

CCAGCAG

MVE-KO #31 allele 1🡪 1bp insertion + 48bp deletion🡪 frame shift

ATGGTCTACGATGACACCAGTAA**A**GAAGTGGGTACCGATCAAGCCTGGCCAGCAGGGATTCAGCCGGATCAACATCTACCACAACACTGCCAGCAGCACCTTCAGAGTGGT------------------------------------------------ACGTACCC

MVE-KO #31 allele 2🡪 3bp deletion 1bp insertion 🡪 frame shift

ATGGTCTACGATGACACCAGTAA---GTGGGTACCGATCAAGCCTGGCCAGCAGGGATTCAGCCG

GATCAACATCTACCACAACACTGCCAGCAGCACCTTCAGAGTGGTCGGGGTCAAGC**G**TACAGGACCAGCAG

**NIH 3T3 fibroblasts**

**NIH 3T3 WT (Mena target sequence)**

CTACACAGACTTTCCACCAATGGAGGGATGCTAGACAGGTGTATGGTCTCAACTTTGGCAGCAAAGAGGATGCCAATGTCTTCGCAAGTGCCATGATGCATGCCTTAGAAGTGTTAAATTCACAGGAAGCAGGTAAAGTGTCTGTGCTTTCCCAGTACTCTGCAGTGGAAATGCTATGTATATGTTAAAGCAAAACTTTCTCCTCAGCTTTTAAGTCATATATATATATTTTAAATAGTTCCTGTTACTTTCTAGAAAAATGTGAACTCTTTCTTACTTGTTTGTGTTAAAACTATAGTCTATCTGGGCATGGTG

Tide analysis M-KO #10


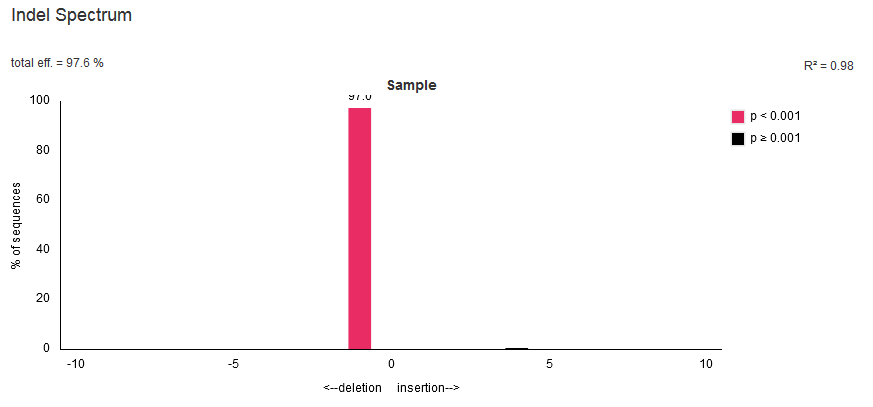


M-KO #10 alleles 1 and 2 🡪 1 bp deletion 🡪 frame shift

CTACACAGACTTTCCNCNCAATGGAGGGATGCTAGACAGGTGTATGGTCTCAACTTTGGCAGCAAAGAGGATGCCAATG-CTTCGCAAGTGCCATGATGCATGCCTTAGAAGTGTTAAATTCACAGGAAG

CAGGTAAAGTGTCTGTGCTTTCCCAGTACTCTGCAGTGGAAATGCTATGTATATGTTAAAGCAAAACTTTCTCCTCAGCTTTTAAGTCATATATATATATTTNAAATAGTTCCTGTTATTTTCTAGAAAAATGTGAACTCTTTCTTACTTGTTTGTGTTAAAACTATAGTCTATCTGGGCATG

Tide analysis M-KO #17


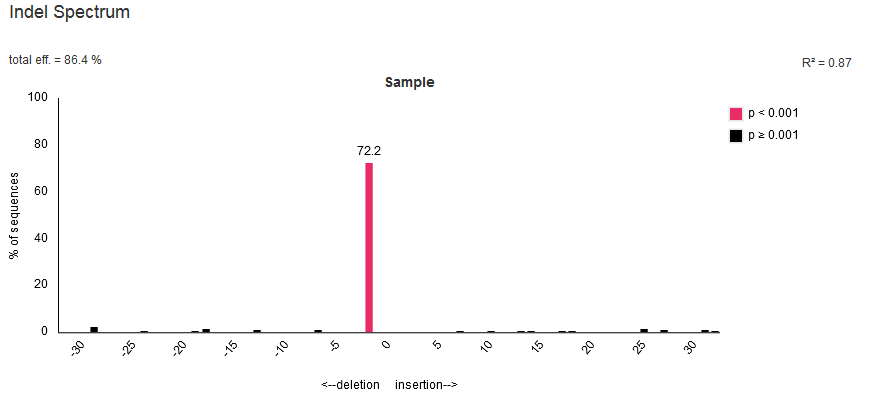


M-KO #17 alleles 1 and 2 🡪 1 bp deletion 🡪 frame shift

ANCGAANAGGATGCCAAT-TCTTCGCAAGTGCCATGATGCATGCCTTANAANTGTTAATTCAACA

GGAAGCANGTAAAGTGTCTGNGCTTTCCCAGTACTCTGCAGTGGAAATGCTATGTATATGTTAAAGCAAAACTTTCTCCTCAGCTTTTAAGTCATATATATATATTTAAAATAGTTCCTGTTATTTTCTAGAAAAATGTGAACTCTTTCTTACTTGTTTGTGTTAAAACTATAGTCTATCTGGGCATG

**NIH 3T3 WT (VASP target sequence)**

TTGGGGTTTCTAGTCAGGGTCTCTAACCACTGAGCAACACTCCCAGTCATGCATGTACAGCCACACCCCTGTATATTCATGTACAGGCTGCAATCAGTTCCTGTATCTACTATATGGATCTAAGGAGAATGTGACTGGCCCCTCTGTTTTCTCCCCTGGCAGCGAGACGGTCATCTGTTCCAGCCGGGCTACTGTGATGCTTTATGATGACAGCAACAAGCGATGGCTCCCTGCTGGCACTGGTCCCCAGGCCTTCAGCCGCGTCCAGATCTACCACAACCCCACTGCTAACTCCTTCCGAGTTGTTGGCCGCAAGATGCAGCCGGATCAGCAGGTGCAGGCTCCCTCTGTCCCTC

Tide analysis M-KO #10.6


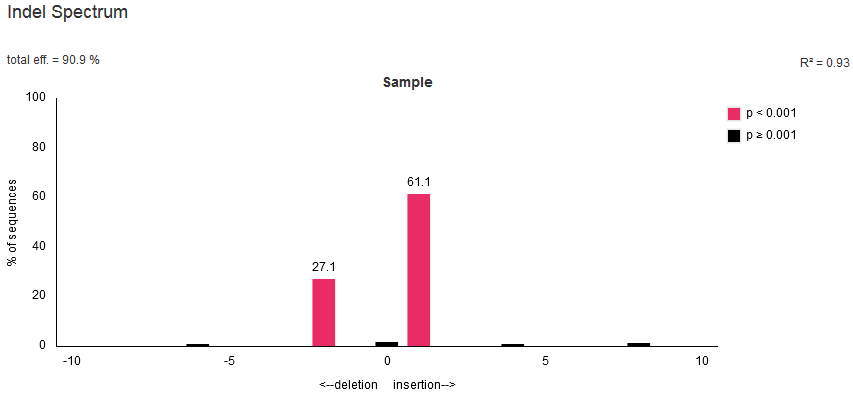


MV-KO #10.6 allele 1 🡪 2 bp deletion🡪 frame shift

TTGGGGTTTCTAGTCAGGGTCTCTAACCACTGAGCAACACTCCCAGTCATGCATGTACAGCCACACCCCTGTATATTCATGTACAGGCTGCAATCAGTTCCTGTATCTACTATATGGATCTAAGGAGAATGTGACTGGCCCCTCTGTTTTCTCCCCTGGCAGCGAGACGGTCATCTGTTCCAGCCGGGCTACTGTGATGCTTTATGATGACAGCAACAAGCGATGGCTCCCTGCTGGCACTGGTCCCCAGGCCTTCA--C

GC

MV-KO #10.6 allele 2 and 3 🡪 1 bp insertion 🡪 frame shift

TTGGGGTTTCTAGTCAGGGTCTCTAACCACTGAGCAACACTCCCAGTCATGCATGTACAGCCACACCCCTGTATATTCATGTACAGGCTGCAATCAGTTCCTGTATCTACTATATGGATCTAAGGAGAATGTGACTGGCCCCTCTGTTTTCTCCCCTGGCAGCGAGACGGTCATCTGTTCCAGCCGGGCTACTGTGATGCTTTATGATGACAGCAACAAGCGATGGCTCCCTGCTGGCACTGGTCCCCAGGCCTTCA**C**GCCGCGTCAAGATCTACCACAACCCCGCTGCTAACTCCNTNNNAGTTGTTGGCCGCAAGATGCANNC

Tide analysis M-KO #10.9


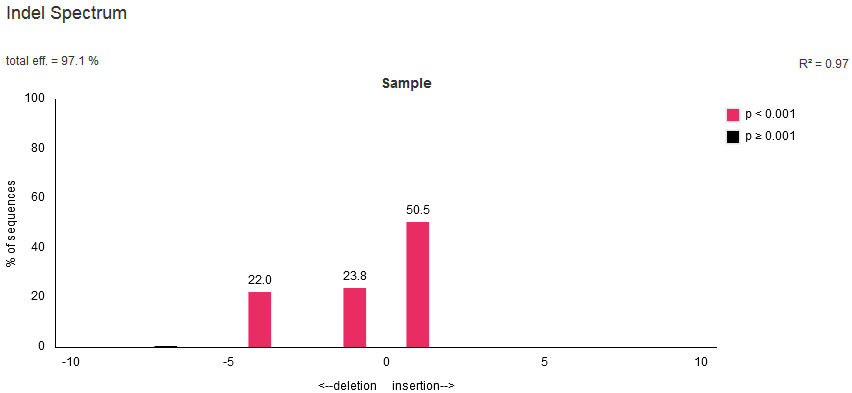


MV-KO #10.9 allele 1🡪 4 bp deletion 🡪 frame shift

TCTAGTCAGGGTCTCTAACCACTGAGCAACACTCCCAGTCATGCATGTACAGCCACACCCCTGTATATTCATGTACAGGCTGCAATCAGTTCCTGTATCTACTATATGGATCTAAGGAGAATGTGACTGGCCCCTCTGTTTTCTCCCCTGGCAGCGAGACGGTCATCTGTTCCAGCCGGGCTACTGTGATGCTTTATGATGACAGCAACAAGCGATGGCTCCCTGCTGGCACTGGTCCCCAGGCCTTCA----CGCCGCG

ACC

MV-KO #10.9 allele 2🡪 1 bp deletion

TCTAGTCAGGGTCTCTAACCACTGAGCAACACTCCCAGTCATGCATGTACAGCCACACCCCTGTATATTCATGTACAGGCTGCAATCAGTTCCTGTATCTACTATATGGATCTAAGGAGAATGTGACTGGCCCCTCTGTTTTCTCCCCTGGCAGCGAGACGGTCATCTGTTCCAGCCGGGCTACTGTGATGCTTTATGATGACAGCAACAAGCGATGGCTCCCTGCTGGCACTGGTCCCCAGGCCTTCA-CGCCGCGACC

MV-KO #10.9 alleles 3 and 4🡪 1 bp insertion 🡪 frame shift

TAGTACTGTGGATTCCNACCAAGGGCCCGGGCCAGCAAGCTGGGCAAACACGCTACTATTGAGCCACACCCCCAGCCCCTCATTGGGGTTTCTAGTCAGGGTCTCTAACCACTGAGCAACACTCCCAGTCATGCATGTACAGCCACACCCCTGTATATTCATGTACAGGCTGCAATCAGTTCCTGTATCTACTATATGGATCTAAGGAGAATGTGACTGGCCCCTCTGTTTTCTCCCCTGGCAGCGAGACGGTCATCTGTTCCAGCCGGGCTACTGTGATGCTTTATGATGACAGCAACAAGCGATGGCTCCCTGCTGGCACTGGTCCCCAGGCCTTCA**C**GCCGCGTCAAGATCTACCACAACCCCGCTGCTAACTCCNTNNNAGTTGTT
